# Enhancing the Interpretability of Malaria and Typhoid Diagnosis with Explainable AI and Large Language Models

**DOI:** 10.3390/tropicalmed9090216

**Published:** 2024-09-16

**Authors:** Kingsley Attai, Moses Ekpenyong, Constance Amannah, Daniel Asuquo, Peterben Ajuga, Okure Obot, Ekemini Johnson, Anietie John, Omosivie Maduka, Christie Akwaowo, Faith-Michael Uzoka

**Affiliations:** 1 Department of Mathematics and Computer Science, Ritman University, Ikot Ekpene 530101, Nigeria; eke5461@gmail.com (E.J.); aniettejohn5@gmail.com (A.J.); 2 Department of Computer Science, Faculty of Computing, University of Uyo, Uyo 520103, Nigeria; mosesekpenyong@uniuyo.edu.ng (M.E.); okureobot@uniuyo.edu.ng (O.O.); 3 Science, Technology, Engineering and Mathematics (STEM) Centre, University of Uyo and Centre for Research, University of Uyo, Uyo 520103, Nigeria; 4 Department of Computer Science, Ignatius Ajuru University of Education, Port Harcourt 500102, Nigeria; aftermymsc@gmail.com; 5 Department of Information Systems, Faculty of Computing, University of Uyo, Uyo 520103, Nigeria; danielasuquo@uniuyo.edu.ng; 6 Department of Computer Engineering, Faculty of Engineering, Gregory University, Uturu 441106, Nigeria; ajugapeterben@gmail.com; 7 University of Port Harcourt Teaching Hospital, Port Harcourt 500102, Nigeria; omosivie.maduka@gmail.com; 8 University of Uyo Teaching Hospital, Uyo 520103, Nigeria; christieakwaowo@uniuyo.edu.ng; 9 Department of Mathematics and Computing, Mount Royal University, Calgary, AB T3E 6K6, Canada; fuzoka@mtroyal.ca

**Keywords:** malaria diagnosis, typhoid diagnosis, machine learning, XAI, LIME, GPT, BERT, ChatGPT, Gemini, perplexity, explainability, interpretability

## Abstract

Malaria and Typhoid fever are prevalent diseases in tropical regions, and both are exacerbated by unclear protocols, drug resistance, and environmental factors. Prompt and accurate diagnosis is crucial to improve accessibility and reduce mortality rates. Traditional diagnosis methods cannot effectively capture the complexities of these diseases due to the presence of similar symptoms. Although machine learning (ML) models offer accurate predictions, they operate as “black boxes” with non-interpretable decision-making processes, making it challenging for healthcare providers to comprehend how the conclusions are reached. This study employs explainable AI (XAI) models such as Local Interpretable Model-agnostic Explanations (LIME), and Large Language Models (LLMs) like GPT to clarify diagnostic results for healthcare workers, building trust and transparency in medical diagnostics by describing which symptoms had the greatest impact on the model’s decisions and providing clear, understandable explanations. The models were implemented on Google Colab and Visual Studio Code because of their rich libraries and extensions. Results showed that the Random Forest model outperformed the other tested models; in addition, important features were identified with the LIME plots while ChatGPT 3.5 had a comparative advantage over other LLMs. The study integrates RF, LIME, and GPT in building a mobile app to enhance the interpretability and transparency in malaria and typhoid diagnosis system. Despite its promising results, the system’s performance is constrained by the quality of the dataset. Additionally, while LIME and GPT improve transparency, they may introduce complexities in real-time deployment due to computational demands and the need for internet service to maintain relevance and accuracy. The findings suggest that AI-driven diagnostic systems can significantly enhance healthcare delivery in environments with limited resources, and future works can explore the applicability of this framework to other medical conditions and datasets.

## 1. Introduction

Typhoid fever and malaria are two of the most prevalent febrile diseases in the world, presenting serious public health issues, especially in tropical and subtropical areas. Typhoid and malaria are common in these areas due to the high humidity, temperatures, inadequate healthcare facilities, and the shortage of qualified healthcare providers [1]. Despite these diseases being caused by different pathogens and transmitted by diverse vectors, they share several similarities as regards epidemiology, clinical manifestation, and co-infection. Their prevalence is attributed to environmental and healthcare factors, including a warm and humid climate, rapid urbanization without adequate infrastructure, which results in crowded living conditions and poor sanitation, limited access to high-quality healthcare, a lack of preventive measures, and weak disease surveillance systems in these regions. Typhoid fever and malaria continue to be the leading causes of morbidity and mortality [2]. *Salmonella enterica* serotype Typhi is the bacteria that causes typhoid fever or enteric fever, which affects millions of people worldwide and can have serious consequences if left untreated [3,4,5]. Malaria, on the other hand, is caused by *plasmodium* parasites that are transmitted by *Anopheles* mosquito bites, infecting millions of people and claiming the lives of hundreds of thousands every year [6,7,8]. Malaria remains one of the world’s most serious health problems [9] and the second most studied disease according to a systematic review [10]; this is due to its widespread prevalence, high mortality rate, drug resistance, and environmental factors such as climate change in tropical regions. The prompt and effective diagnosis of these febrile diseases is essential for efficient treatment and care, but current diagnostic techniques often face limitations in accessibility, specificity, and sensitivity. Blood smear examination (microscopy) and rapid diagnostic tests (RDTs) are the current diagnostic techniques for malaria while the Widal test and blood culture are the tests for typhoid fever. Since blood smear microscopy is low-cost, effective, and capable of differentiating between malaria species and quantifying parasites, it is the gold standard for diagnosing malaria. However, it does require a functional infrastructure and skilled, qualified microscopists. RDTs identify malaria antigens in a small volume of blood by using monoclonal antibodies that are directed against the target parasite antigen and impregnated on a test strip but may be less sensitive to identify mixed or non-*Plasmodium falciparum* infections [11]. The Widal test detects typhoid fever in patients’ serum using a suspension of dead *Salmonella enterica* as an antigen. Still, it has low specificity and sensitivity, which can result in incorrect diagnosis and treatment. In contrast, blood culture has high specificity but can have compromised sensitivity due to low bacterial loads or previous antibiotic use [12,13].

Machine learning (ML) algorithms are frequently used in the healthcare sector to help decision-makers make well-informed decisions [14,15]. Medical diagnostics has found ML to be a potent tool that can improve the efficiency and accuracy of diagnosis, but to guarantee that medical professionals can rely on and comprehend the judgments made by these models, the use of ML models in clinical settings demands a high level of interpretability and transparency. Studies have applied numerous ML techniques in diagnosing malaria [16,17,18] and typhoid fever [19], as well as both conditions together [20,21,22]. Even though ML models are frequently used to diagnose diseases, the lack of integrated explainability in previous research makes it difficult for medical professionals to have high confidence in the predictions. According to Anderson and Thomas [23], concerns about ML algorithms’ lack of interpretability frequently impede their acceptance in the healthcare sector. Since the healthcare sector is highly regulated, there is a high demand for accountability and transparency in the decision-making processes for ML models before their adoption [24]. Healthcare practitioners must be able to comprehend and interpret the predictions made by ML models to be used safely as these models are used to supplement clinical decision-making. Their capacity to comprehend and interpret the choices made by ML models is critical in this sector, as decisions can have a significant impact on patient outcomes. To address this challenge, an explainable AI (XAI) technique like Local Interpretable Model-agnostic Explanations (LIME) offers insights into how models arrive at their predictions, thereby promoting trust and aiding in clinical decision-making by healthcare professionals. XAI is becoming increasingly important in the healthcare sector, where making decisions has extremely high stakes because it is challenging for healthcare professionals to trust and comprehend the decisions made by traditional machine learning models. In clinical settings, where comprehension of the reasoning behind a diagnosis is critical for patient safety, regulatory compliance, and ethical considerations, the lack of interpretability may impede the adoption of AI [25]. Therefore, XAI offers solutions to these problems by facilitating AI models’ transparent and intelligible decision-making process. XAI techniques such as LIME are widely utilized to clarify the inner workings of complex models. LIME operates by using an interpretable model local to the prediction to approximate the black-box model. It modifies the input data, tracks how the predictions change as a result, and then fits a straightforward, understandable model to these modified samples [26,27]. In situations where individual case explanations are required, LIME is especially helpful as it helps determine which characteristics are most important for a particular prediction. The interpretability of ML models in the healthcare industry is greatly enhanced by LIME, which allows physicians to better comprehend and rely on AI-driven insights, and their capacity to offer concise, useful explanations improves the usefulness of AI systems in the processes of diagnosis and treatment planning. LIME has been applied in several healthcare settings such as in diagnosing diabetes [28], classification of co-morbidities associated with febrile diseases in children and pregnant women [29], and transparent health predictions [30]. To further improve accuracy and explainability, incorporating large language models (LLMs) into diagnostic processes seems promising in combination with XAI techniques. LLMs are advanced AI systems built using deep learning techniques and trained on vast amounts of data to accomplish a wide range of natural language processing (NLP) tasks. These models can bridge the gap between complex ML algorithms and clinical understanding. They are trained on a wealth of medical data and can provide distinctive interpretations and generate detailed, contextually relevant explanations for diagnostic outcomes.

The use of LLMs in medical contexts has advanced significantly thanks to projects like Generative Pre-trained Transformer (GPT) and Bidirectional Encoder Representations from Transformers (BERT). These models can produce human-like text and comprehend intricate linguistic patterns because they have been trained on enormous volumes of text data. The applications of BERT go beyond identifying pandemic illnesses; it can also be used to process electronic medical records and evaluate the results of goals-of-care talks in clinical trials [30,31,32,33]. GPT has proven to be remarkably adept at producing coherent and contextually relevant text in various domains [34]. GPT can help in the healthcare industry by delivering comprehensive patient reports, producing justifications for medical diagnoses, and offering assistance during clinical decision-making processes [35]. The accuracy and explainability of diagnostic systems can be greatly improved by integrating these LLMs and they can produce thorough narratives that clarify the reasoning behind diagnostic predictions, which facilitates clinician comprehension and validation of AI recommendations. This ability is essential for bridging the gap between cutting-edge AI models and real-world, routine clinical use, raising the standard of healthcare delivery as a whole.

Several other studies have integrated ML and XAI in diagnosis such as predicting the risk of hypertension [36], preventing breast cancer [37], differentiating bipolar disorder [38], predicting hepatitis C [39], and modeling comorbidity in patients with febrile diseases [29]. Other studies have proposed LLMs for healthcare purposes such as the prediction of potential diseases [40], multimodal diagnosis [41], answering cardiology and vascular pathologies questions [42], and answering questions on health diagnosis [43], but there appears to be a gap in the literature regarding the combined use of all three methods (ML, XAI, and LLMs) in diagnosing febrile diseases such as malaria and typhoid fever.

This study aims to enhance the interpretability of typhoid and malaria diagnosis using ML techniques like Extreme Gradient Boost (XGBoost), Random Forest (RF), and Support Vector Machine (SVM) with LIME, and LLMs such as GPT, Gemini, and Perplexity. RF reduces the chance of overfitting and produces a robust result by combining multiple decision trees. The XGBoost algorithm is incredibly scalable and effective, capable of effectively managing both linear and nonlinear relationships while SVM can generalize well to new data, making it a dependable tool for diagnosing diseases with similar symptoms. The XIA tool gives healthcare workers concise explanations for every diagnosis, assisting them in determining which symptoms had the greatest influence on the diagnosis. The LLMs further improve the output and increase the tool’s usability for non-specialists by converting complex technical explanations into plain language. This study emphasizes the potential of integrating these tools to interpret and contextualize medical data, hence bridging the gap between healthcare worker comprehension and complex ML diagnoses. A dataset consisting of patients’ symptoms and diagnoses of malaria and typhoid was collected from healthcare facilities across the Niger Delta region of Nigeria. By leveraging these advanced tools, we seek to develop a diagnostic model that delivers precise diagnoses and provides transparent and understandable insights into their decision-making processes. This research holds significant potential to improve diagnostic practices, ultimately contributing to better patient outcomes and advancing the field of medical diagnostics. This study can advance the field of diagnostic medicine and enhance diagnostic procedures, which will ultimately lead to better patient outcomes. This study’s primary contributions are:

- The consideration of multiple diseases (typhoid fever and malaria) allows for a thorough evaluation of the patient’s health, which is essential for managing co-infection and comorbidity.
- Using real-world data ensures that the models are trained and validated on clinical cases, thereby enhancing the practical relevance and applicability of our findings.
- The black-box nature of many ML models is addressed by the integration of an XAI method, which gives medical professionals transparent and comprehensible insights into how each feature influences the diagnosis, ensuring that diagnostic results are presented in a way that is meaningful for easier interpretation. By focusing on interpretability, healthcare workers can make more accurate and timely diagnoses.
- LLMs give the diagnosis process an extra layer of context-aware understanding and incorporating them makes it possible to better understand and analyze complex medical outcomes.
- The combination of LLMs and conventional ML models enables a thorough comparison of various diagnosis strategies. This not only demonstrates the models’ efficacy but also the advantages and disadvantages of each approach to managing medical data.
- The integration of XAI, LLMs, and ML puts this work at the forefront of medical AI research. It demonstrates the viability and benefits of using a hybrid approach to address difficult diagnostic problems, establishing a standard for further study in the area.

The study is prepared as follows: The methodology is presented in Section 2, including data collection, preprocessing, and the application of XAI and ML models, along with the incorporation of LLMs for improved diagnostic interpretability. The results are discussed in Section 3, evaluating the effectiveness of various algorithms and illustrating how XAI offers insights into model decisions, along with the implications for clinical practice. Section 4 concludes the study, highlighting its limitations, and offering recommendations for further research to advance diagnostic techniques.

## 2. Methodology

### 2.1. Malaria and Typhoid Diagnosis Framework

**Figure 1:**
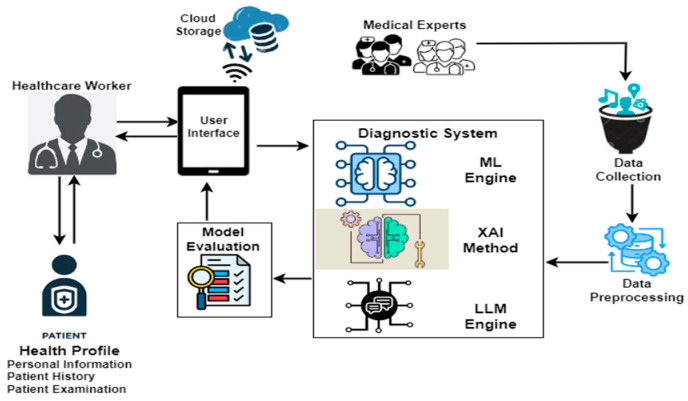
Malaria and Typhoid Fever Diagnosis Framework.

The proposed diagnosis framework for malaria and typhoid fever is presented in Figure 1. The major components of the framework include a healthcare worker, medical experts, and a mobile device for the collection, processing, and storage of information locally and on a cloud-based storage for decision making. Patient data were obtained from medical experts and pre-processed into a format suitable for machine learning modeling and processing by large language models. Pre-processing ensures data quality, selects and encodes pertinent features, balances the dataset, and normalizes inputs, which contributes to the model’s ability to make more dependable predictions. The proposed model can be utilized in the diagnoses of typhoid fever and malaria with enhanced accuracy and explainability by a healthcare worker through a mobile device. Through the user-friendly interface, healthcare workers can input patient’s vitals and symptoms using dropdown menus and sliders. After the data are entered, the model can process them and instantly diagnose the patient as likely having typhoid fever, malaria, neither, or both.

### 2.2. Description of the Dataset Used for the Study

The New Frontiers in Research Fund project’s dataset instrument, designed by a team of medical experts in the field of febrile diseases and computer scientists, was used in this study. The dataset, comprising 4870 patient records, was organized into six sections, including demographic data, patient symptoms, risk factors, and diagnosis information [44]. The first section contains the patient demographics as shown in Table 1 and the diagnosing physician’s information. The second section contains the patient’s symptoms on a five-point scale (1 = absent; 2 = mild; 3 = moderate; 4 = severe; 5 = very severe), along with the doctor’s level of confidence (a numerical rating scale from 1 to 10). The five-point symptom scale is based on clinical reality, where symptoms vary in severity, and it ensures that data collection is consistent across various doctors and cases, reducing variability and potential bias. The patient’s degree of susceptibility to the other non-clinical risk factors was listed in the third section, and the doctor’s initial diagnosis was listed in the fourth. The confirmed diagnosis was included in the sixth section of the dataset after further investigations such as full blood count, blood film, and serology were conducted on the patient in the fifth section. A linguistic scale (1 = absent; 2 = very low; 3 = low; 4 = moderate; 5 = high; 6 = very high) was used to rate the intensity of attack for both preliminary and confirmed diagnoses (Sections 4 and 6), along with the doctor’s degree of confidence (1–10) in each case. The dataset contained malaria, typhoid, HIV, respiratory tract infection, urinary tract infection, tuberculosis, lassa fever, yellow fever, and dengue fever with a total of 50 symptoms.

### 2.3. Data Preprocessing and Oversampling

The collected dataset comprised columns with both numeric and string data types, along with a few missing values. Missing values are a common problem in datasets and can cause bias, reduce model accuracy, and complicate data preprocessing, all of which can negatively affect ML model performance.

Data preprocessing was conducted, including feature selection, feature scaling, and data cleaning. Records with missing features, irrelevant data, and columns that were not needed were eliminated during the data-cleaning process. Records with missing symptoms were likewise eliminated to maintain the integrity of the dataset. Because the patient consultation tool did not include symptoms for patients under the age of five, records of those patients were removed. This is because patients in this age group may not be able to accurately express certain symptoms, leaving them to rely entirely on their parents’ interpretation. A selection of the most pertinent and significant features for modeling febrile illness (malaria and typhoid fever symptoms) was made to carry out the feature selection process. The dataset was reduced to 3914 records, including only the malaria and typhoid fever confirmed diagnoses and their twelve (12) symptoms. These two diseases with their 12 symptoms were selected from the list of symptoms because the rest of the diseases were underrepresented in the dataset, leading to an imbalanced dataset. The scope was narrowed to these two diseases to enhance the model’s ability to detect and differentiate between these two diseases more effectively.

**Figure 2:**
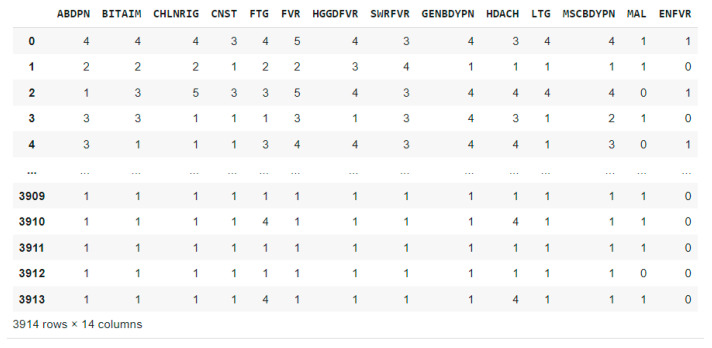
Pre-processed dataset.

A patient’s symptoms, the intensity of each symptom, and confirmed diagnoses (malaria and typhoid fever) are all included in the processed dataset. The list of symptoms and diseases with abbreviations is presented in Table 2. As shown in Figure 2, custom mapping was used to map the disease severity ‘Absent’ (1) to binary 0 and ‘Very-low’ to ‘Very-severe’ (2 to 6) to binary 1.

**Figure 3:**
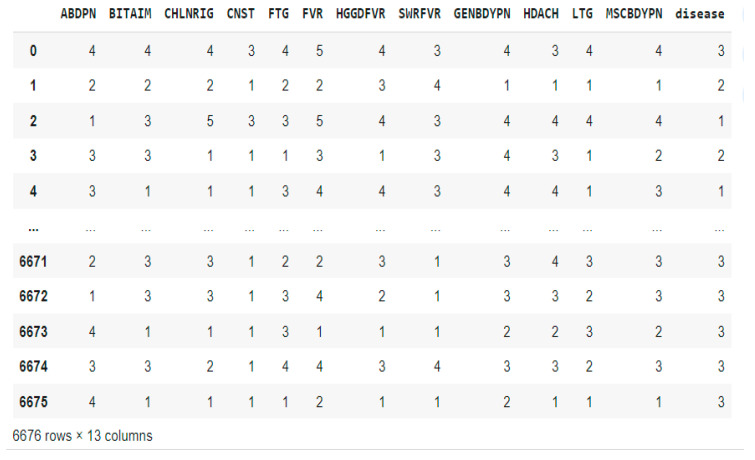
Oversampled dataset with SMOTE.

After further analysis, we noticed that of the 3914 patients, 1088 patients had neither malaria nor typhoid fever, 1669 had only malaria, 107 had only typhoid fever, and 1050 had both diseases. The Synthetic Minority Oversampling Technique (SMOTE) was employed to handle the class imbalance. SMOTE has several advantages and when compared to just replicating minority class instances, it lowers the chance of overfitting by creating synthetic samples. It improves model performance, is compatible with most ML techniques, and is useful for various types of data. SMOTE identified minority class instances, selected k-nearest neighbors, and generated and added synthetic samples to the original dataset, as presented in Figure 3. The oversampled dataset contains 6676 patient records with the class labels 0 (No disease), 1 (Typhoid only), 2 (Malaria only), and 3 (Both diseases) in the ‘Disease’ column.

### 2.4. Diagnostic Models and Model Optimization

We used Google Colaboratory (Colab), a free cloud-based platform from Google that offers a Python programming environment with quick access to robust graphics processing unit (GPU) resources and ML libraries. Additionally, the platform provides a CPU runtime and easily integrates Google Drive for storage. Python packages and libraries such as NumPy, Pandas, Scikit-Learn, and Matplotlib, which are necessary for creating classification models, were utilized. The ML algorithms used in building our diagnostic models and the performance metrics are presented in the subsection incorporating hyperparameter tuning, known as grid search cross-validation (GridSearchCV), which is used to increase the precision of the diagnosis. GridSearchCV is an expanded method for optimizing hyperparameters by enabling customized search spaces for each hyperparameter, using designated ranges. The hyper-parameter setting used was: SVM (‘C’: [0.1, 1, 10, 100], ‘gamma’: [‘scale’, ‘auto’, 0.001, 0.01, 0.1], ‘kernel’: [‘rbf’, ‘linear’]). ‘C’ is the parameter that controls the trade-off between achieving a low error on the training data and minimizing the model complexity, Gamma defines how far the influence of a single training example reaches, while the Kernel function transforms the data into a higher-dimensional space to make them easier to separate using a linear boundary. XGBoost (‘max_depth’: [3, 4, 5, 6], ‘learning_rate’: [0.01, 0.1, 0.2], ‘n_estimators’: [100, 200, 300], ‘colsample_bytree’: [0.3, 0.7]), where max_depth determines the maximum depth of the trees, learning_rate controls how much the model’s weights are adjusted to the loss gradient, n_estimators indicate the number of trees to be built, and colsample_bytree defines the subsample ratio of columns when constructing each tree. RF(‘n_estimators’: [100, 200, 300], ‘max_depth’: [None, 10, 20, 30], ‘min_samples_split’: [2, 5, 10], ‘min_samples_leaf’: [1, 2, 4], ‘bootstrap’: [True, False]), where min_samples_split determines the minimum number of samples required to split an internal node, min_samples_leaf specifies the minimum number of samples required to be at a leaf node, and bootstrap determines whether bootstrap samples are used when building trees. Each of these hyperparameters aids in fine-tuning the behavior of the model, enhancing its functionality and ability to diagnose the febrile illnesses considered in this study with good generalization. These hyperparameters were derived from built-in functions of the corresponding ML algorithms. Our local laptops utilized for this study were a Dell Latitude 7480, Core i5-7200U CPU @ 2.50 GHz (4 CPUs), ~2.7 GHz with 16 GB RAM for the ML and XAI modeling while a Samsung 950QDB, Core i7-1165G7 @ 2.80Ghz (8 CPUs) ~ 2.8 GHz with 16 GB RAM was used for the large language modeling. We used Visual Studio Code, a free coding editor that supports several extensions and allows for quick coding initiation. LLMs are easily accessible thanks to the Python foundation of our development environment. The process was automated by utilizing core Python packages and libraries, such as pandas, numpy, flet, matplotlib, flask, flask-sqlalchemy, seaborn, sk-learn, and joblib for loading models. The information extractor comprises a prompt generator, automator, and interpreter. The Malaria and Typhoid Diagnosis System interacts with various application programming interfaces (APIs) for database communication and diagnosis management. It has two main components: the front-end, built using Flet with Matplotlib and Seaborn for visualizations, and the back-end, powered by Flask for API integration and MySQL database management via Flask-SQLAlchemy. The prompt generator converts data into a readable format, saves prompts in a JSON file, and organizes the patient’s symptoms and severity into manageable prompts. The prompt used by Caruccio et al. [45] mimics the conversation of a physician when seeking assistance in diagnosing a patient based on particular symptoms. The template is “The patient has these symptoms: [S] Tell me which of the following diagnoses is most related to the symptoms: [D]? [H]”. Where [S] is all of the symptoms listed in the prompt, [D] the diagnoses the LLM must decide on, and [H] the answer or diagnoses provided by the LLM. This template was modified to arrive at our prompt: “The patient has these symptoms with severity levels, listed in the table below. (create a table with only the diagnosis column filled in), the output should be in CSV format, diagnosis [Malaria, Typhoid Fever, Both, None]?”. The automator manages data flow by retrieving outputs and storing them in a JSON file. It feeds these prompts into the large language models (GPT, Gemini, and Perplexity). After that, the interpreter transforms the JSON output into an Excel file so that reporting and analysis of the data can be carried out. The link to the scripts can be found in this GitHub account https://github.com/FebrileDiseasesDiagnoses/Auto_tool.git (2 August 2024).

#### 2.4.1. Random Forest

**Figure 4:**
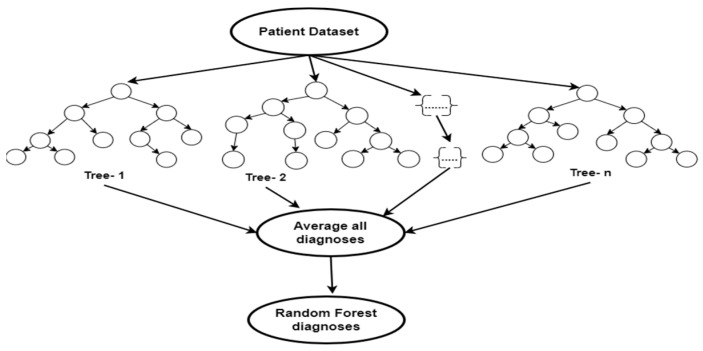
Random Forest schematic diagram.

Random Forest algorithm is an ensemble ML technique with robust resistance to over-fitting that combines several decision trees to increase prediction accuracy [46], as shown in Figure 4. RF trains predictions concurrently, operates well on large datasets, and is good at estimating missing data [47]. RF can easily resolve high-dimensional and complex problems such as the prediction of disease conditions [48,49,50]. By combining individual tree predictions via voting, the final prediction is produced. This approach increases the model’s robustness, decreases overfitting, and can aid in diagnosing febrile diseases.

#### 2.4.2. Extreme Gradient Boost

**Figure 5:**
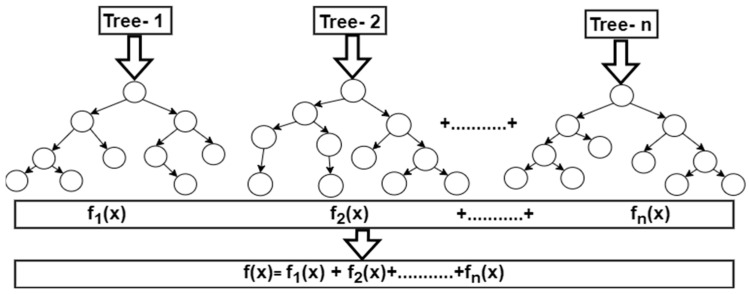
Extreme gradient boosting schematic diagram.

XGBoost algorithm is a component of the gradient boosting framework, which can be applied to regression or classification predictive modeling issues. Figure 5 depicts the computation process used by XGBoost as it introduces weak learners into the ensemble, focusing each new learner on correcting the mistakes made by the previous ones. Because of its reputation for managing structured data, XGBoost is extensively utilized in numerous applications, including the prediction of disease [51].

#### 2.4.3. Support Vector Machine

**Figure 6:**
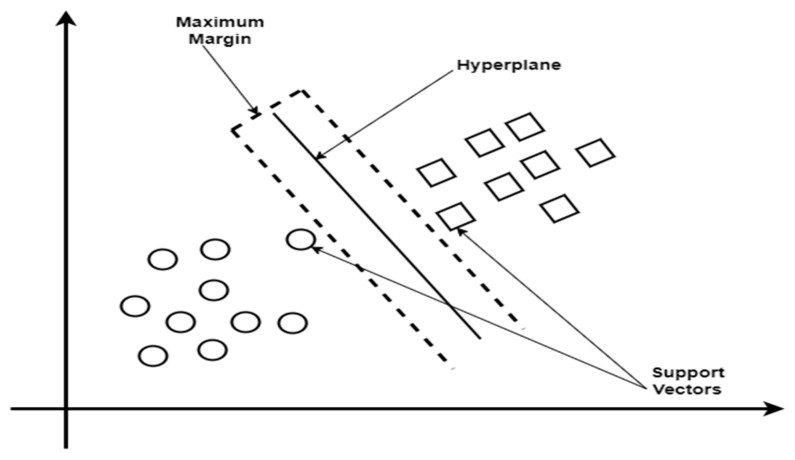
Support Vector Machine diagram.

SVM is well-known for working well in high-dimensional spaces and for handling non-linearly separable data by utilizing kernel functions. It seeks to determine which hyperplane best divides the data into distinct classes. The margin is the distance between the hyperplane and the closest observations, and the support vectors are the points that are closest to it, as shown in Figure 6. SVM uses little memory, performs well with a wide range of features, and can be tailored with various kernel functions for intricate decision boundaries. SVM is resistant to overfitting and can handle high-dimensional data as well as binary and multi-class classification issues in medical diagnosis, making it an effective tool for diagnosing diseases [52].

### 2.5. Interpretability and Explainability Methods

Local Interpretable Model-agnostic Explanations (LIME) approximates the complex model near a particular prediction with an interpretable model such as a linear model to provide local explanations. The integration of LIME into our model follows these key steps: (i) Instance Selection: LIME was applied to each instance in the test dataset, generating localized explanations for the model’s predictions on a case-by-case basis; (ii) Feature Contribution Analysis: LIME produces visualizations that indicate the contribution of each feature to the prediction. Features that positively influence the likelihood of a specific disease are displayed on the right side of the plot, while those that decrease the likelihood are shown on the left; (iii) Global Insight Aggregation: By aggregating LIME explanations across multiple instances, we can identify patterns and key features that consistently influence the model’s decisions, providing a broader understanding of the model’s behavior across the dataset.

Generative Pre-trained Transformer (GPT) is pre-trained using unsupervised learning on a large corpus of text data, where it learns to predict the word that will appear next in a sequence based on every word that has come before it. This pre-training gives GPT a thorough grasp of language syntax, semantics, and context. When GPT is fine-tuned on particular tasks, like text generation, question answering, or text completion, it uses its learned representations to produce outputs that make sense and are relevant to the context. GPT is an effective tool for NLP applications because it can produce text similar to that of a human being and manage a wide range of linguistic tasks.

Bidirectional Encoder Representations from Transformers (BERT) is a transformer-based model that is trained to predict missing words in both directions with the help of masking certain words in the input and making predictions about them using both left and right context. Thanks to this bidirectional training, it can capture more complex contextual meanings and relationships within text, producing more accurate language representations. BERT can comprehend subtleties in language and performs well on a variety of natural language understanding tasks, including named entity recognition, sentiment analysis, and machine translation, thanks to its large-scale pre-training tasks. BERT is a flexible and potent model for a range of NLP applications. Its performance can be further improved by fine-tuning it for particular tasks.

### 2.6. Model Performance Metrics

The dataset used for this study initially contained 4870 patient records with symptoms of febrile diseases. After preprocessing, the records were reduced to 3914, and after oversampling, 6676 patient records with relevant features were retained for ML modeling. In total, 80% of the dataset was used for training and 20% for testing. GridSearchCV was employed to optimize model performance and StratifiedKFold was used for cross-validation, dividing the dataset into five stratified folds and shuffling the data before splitting to ensure robust and unbiased results. The experimental models were evaluated using key performance metric components. True Positives (TP) are cases where the model correctly predicts the positive class, represented by the diagonal elements of the matrix, while True Negatives (TN) are cases where the model correctly predicts the negative class. TN is the sum of all the cells that are neither in the row nor the column corresponding to the class being considered. False Positives (FP) are cases where the model incorrectly predicts the positive class while False Negatives (FN) are cases where the model incorrectly predicts the negative class. When evaluating the sensitivity and specificity of diagnostic tests, these metric components are helpful. The evaluation metrics listed below were used in this paper.

*Accuracy* is a measurement of how well a model predicts all labels linked to each data point in a dataset. Datasets with a balanced distribution of positive and negative samples are good candidates for accuracy. For unbalanced datasets, it is less helpful because they can be deceptive.

(1) $$Accuracy=\frac{True\;Positives+True\;Negatives}{True\;Positives+True\;Negatives+False\;Positives+False\;Negatives}$$

*Precision* is a metric that expresses how accurately a model predicts positive outcomes; it measures the model’s capacity to correctly identify true positive instances while avoiding false positives. When false positives come at a high cost, accuracy matters. In the context of medical diagnosis, for instance, a false positive may result in needless treatments.

(2) $$Precision=\frac{True\;Positives}{True\;Positives+False\;Positives}$$

*Recall* is a metric used to assess a model’s capacity to locate every positive instance in a dataset. It measures how sensitive the model is to True Positive cases. When the cost of false negatives is significant, as in medical screenings, recall is crucial because it can be crucial to miss a positive case (false negative).

(3) $$Recall=\frac{True\;Positives}{True\;Positives+False\;Negatives}$$

*F1-Score* is a metric that represents the harmonic mean of Rrecall and Pprecision. The F1-score is limited to a range of binary values, where 1 denotes that every class’s data point was correctly predicted and 0 denotes that any class’s data point was incorrectly predicted. When you must strike a balance between Rrecall and Pprecision, the F1 Score can be helpful, particularly when your class distribution is not uniform.

(4) $$F1=2\left(\frac{Precision*Recall}{Precision+Recall}\right)$$

Log Loss is a measure of the probability of a prediction’s accuracy and it penalizes the difference between the expected probabilities and the actual class labels. Log loss is helpful when one needs a metric that can handle probabilistic model outputs and penalizes incorrect predictions more severely.

(5) $$Logloss=-\frac{1}{N}\sum_{i=1}^{N}\left[y_{i}\log\left(p_{i}\right)+(1-y_{i})log(1-p_{i})\right]$$

where N is the total number of samples in the dataset, $y_{i}$ is the actual label of the i−th instance, $p_{i}$ is the predicted probability of the i−th instance being in the positive class, and $\log\left(p_{i}\right)$ is the natural logarithm of the predicted probability for the positive class

## 3. Results and Discussion

**Figure 7:**
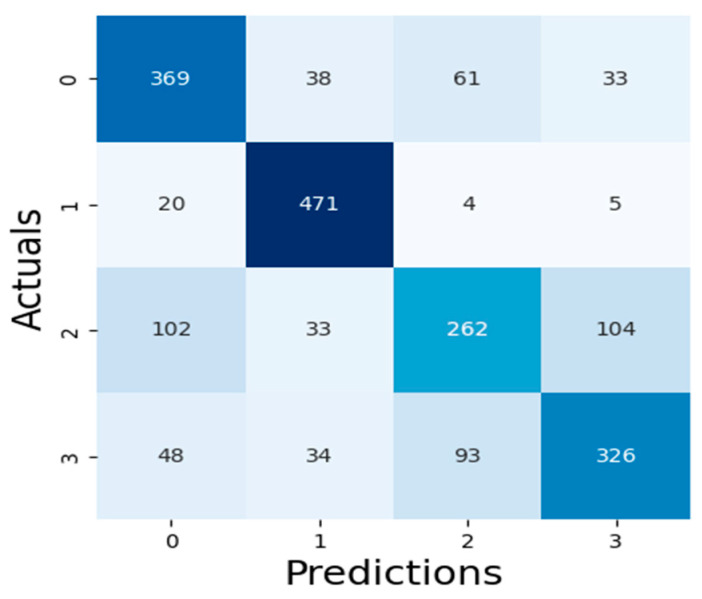
XGBoost Algorithm Confusion Matrix.

**Figure 8:**
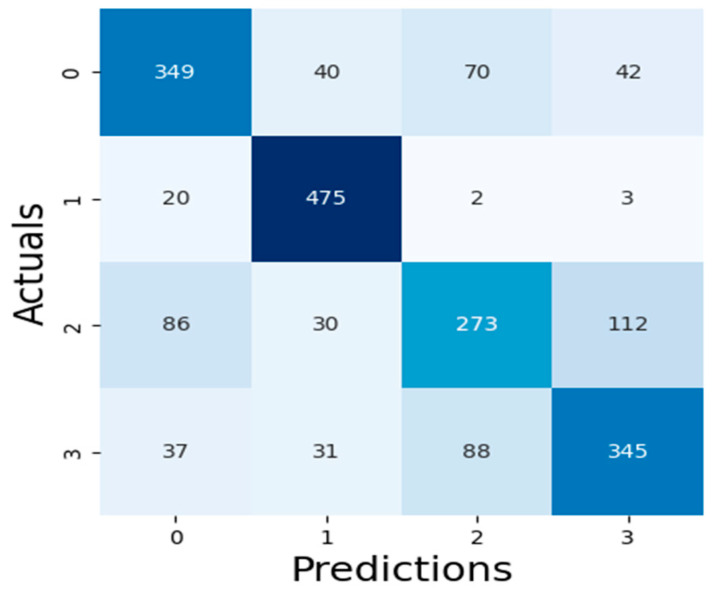
RF Algorithm Confusion Matrix.

**Figure 9:**
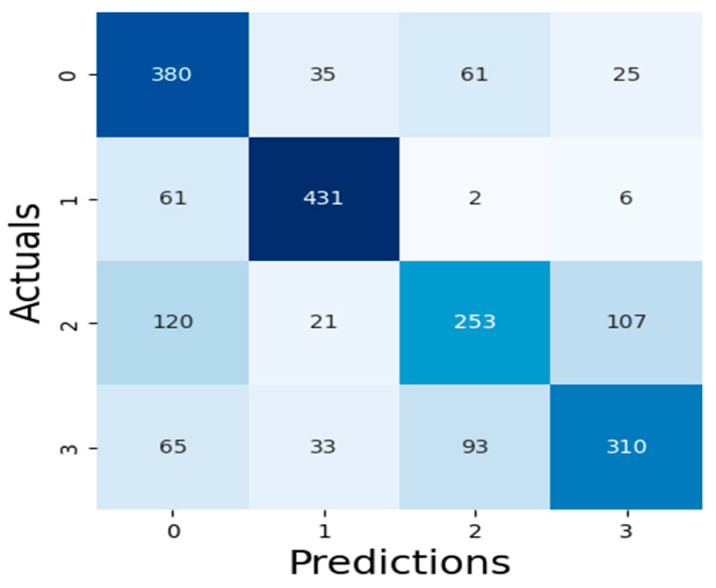
SVM Algorithm Confusion Matrix.

The results of our assessment of the models’ performance are shown in this section, including the XAI method adopted as well as the experimental assessment of the LLMs of Malaria and Typhoid Fever diagnoses. Figure 7, Figure 8 and Figure 9 present the confusion matrices, an essential instrument for assessing how well a classification ML model performs.

**Figure 10:**
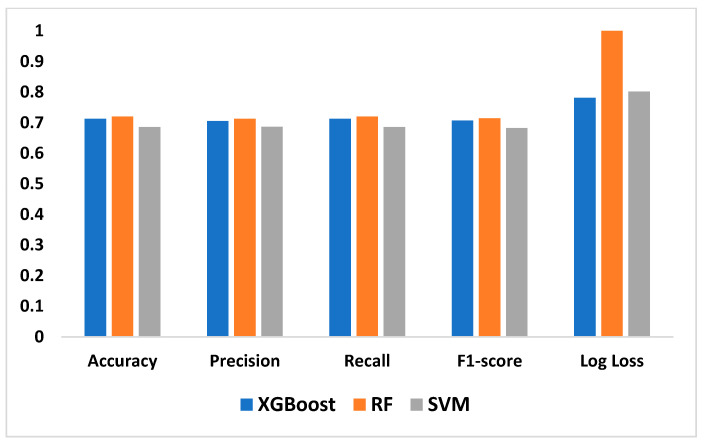
Performance Evaluation of the Machine Learning Models.

Table 3 presents values of these metrics and the computation time of each model while Figure 10 is a pictorial representation of the model’s performance based on the considered metrics. The result shows that RF (accuracy = 71.99%, precision = 71.29%, recall = 71.99%, F1-Score = 71.45%) demonstrates superior performance, outperforming XGBoost (accuracy = 71.29%, precision = 70.56%, recall = 71.29%, F1-Score = 70.66%) and SVM (accuracy = 68.60%, precision = 68.65%, recall = 68.60%, F1-Score = 68.21%). High recall and precision are essential for diagnosing diseases like typhoid and malaria by guaranteeing that the majority of real cases are identified. In this case, high precision helps prevent needless treatments for illnesses that are not present. Because both XGBoost and RF do a good job of balancing these metrics, they are better suited for clinical applications where false positives and false negatives can have detrimental effects. Also, XGBoost has a smaller log loss, which suggests more accurate and well-calibrated probability estimates as well as stronger diagnosis confidence. This may be critical in medical diagnostics, where accuracy is not as important as confidence in the presence of a disease. In medical scenarios where treatment decisions are influenced by the certainty of a diagnosis, lower log loss values for XGBoost indicate that its probability estimates are more reliable. Because of RF’s higher log loss, probability estimates are less trustworthy, which could cause uncertainty when making decisions. SVM performs worse than the other two models in terms of performance metrics and computation time (running time exceeds one hour), implying that it might not work as well for diagnosing typhoid and malaria in this specific dataset. Therefore, ensemble techniques (XGBoost and Random Forest) may be better at capturing the intricate relationships between symptoms and diseases than the SVM model. RF combines the predictions of multiple decision trees to make a final prediction, which results in a slightly higher accuracy but at the cost of increased computational complexity, while XGBoost optimizes each tree by minimizing errors from the previous ones, leading to faster convergence and efficient model optimization. The moderate F1-scores in these models are a result of typhoid fever and malaria having very similar symptoms, making it challenging for the models to differentiate between the two illnesses. This overlap can impair the model’s predictive accuracy, especially concerning recall and precision.

**Figure 11:**
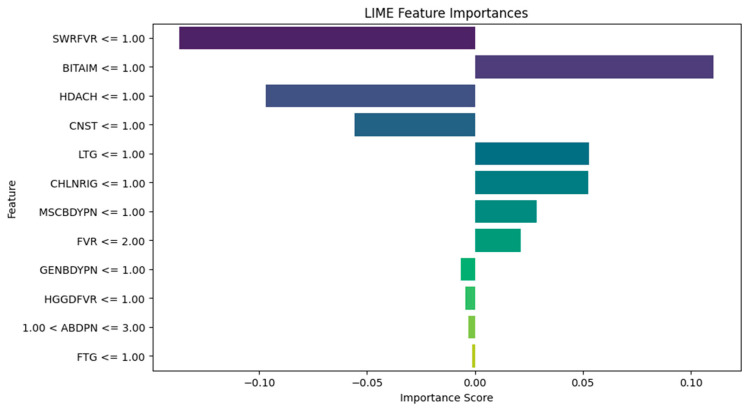
XGBoost Algorithm LIME diagram.

**Figure 12:**
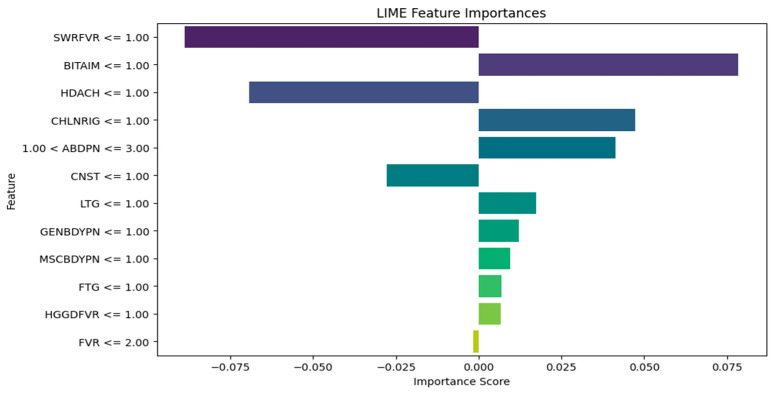
RF Algorithm LIME diagram.

**Figure 13:**
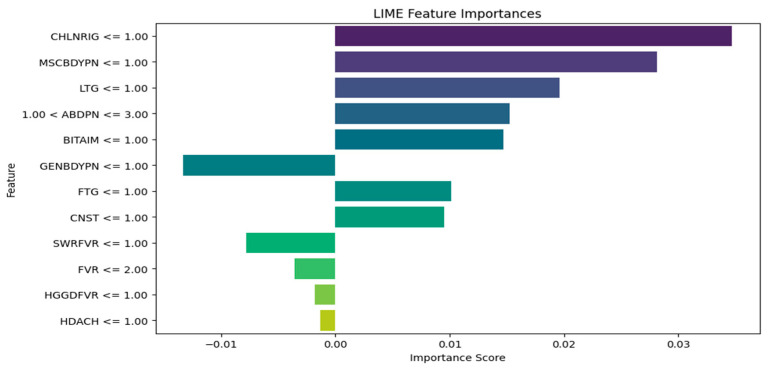
SVM Algorithm LIME diagram.

The LIME plots (Figure 11, Figure 12 and Figure 13) provide a global view of how the features (symptoms) contribute to the model’s diagnoses across the entire test dataset, identifying features with the highest average contributions, both positively and negatively, across all diagnoses. The XGBoost LIME diagram in Figure 11 shows symptoms such as SWRFVR, HDACH, and CNST, as specified by their negative contributions on the left side of the plot, suggesting that the lower levels or absence of these symptoms are associated with a lower likelihood of a patient having malaria and typhoid. Meanwhile, symptoms such as BITAIM, LTG, CHLNRIG, MSCBDYPN, and FVR are the most influential symptoms constantly contributing to the diagnoses of malaria and typhoid across numerous patients.

The RF LIME diagram in Figure 12 also points out that the same symptoms (SWRFVR, HDACH, and CNST) are associated with a lower likelihood of having malaria and typhoid, whereas BITAIM, CHLNRIG, ABDPN, LTG, GENBDYPN, MSCBDYPN, FTG, and HGGDFVR are influential symptoms that contribute to the diagnoses of malaria and typhoid among patients.

Figure 13 shows the SVM LIME diagram, indicating that CHLNRIG has the highest feature importance, followed by MSCBDYPN, LTG, ABDPN, BITAIM, FTG, and CNST as the influential symptoms that contribute to the diagnoses of malaria and typhoid among patients. Meanwhile, GENBDYPN, SWRFVR, FVR, HGGDFVR, and HDACH are associated with a lower likelihood of having malaria and typhoid.

It is observed that medical experts should focus on the following influential symptoms for the diagnosis of malaria and typhoid fever in patients: BITAIM, CHNLNRIG, LTG, ABDPN, MSCBDYPN, FVR, GENBDYPN, FTG, and HGGDFVR. This is consistent with the results of Asuquo et al. [53], where GENBDYPN, CHNLNRIG, ABPN, FVR, FTG, and HGDFVR were observable symptoms. LIME has numerous advantages. It explains the individual diagnosis in a form that is relatively easy for humans to comprehend, aiding healthcare workers to understand why a model made a specific diagnosis. LIME can be applied to many ML models and this versatility makes it suitable for various medical diagnostic systems. In addition, LIME is suitable for generating explanations using local approximations [54]. The limitation of LIME is that it is computationally intensive and expensive to generate explanations for individual diagnoses, especially for large datasets and complex models.

Three sets of experiments were conducted to evaluate the performance of ChatGPT, Gemini, and Perplexity in diagnosing malaria and typhoid. In Experiment 1, one prompt at a time was sent to the LLMs for the first 100 patients in the dataset, recording the outputs in a CSV format to see how they performed with a single set of prompts. Experiment 2 involved sending 100 prompts from the first 100 patients in the dataset to the LLMs and storing the outputs in a CSV format to observe their responses to a series of prompts. In Experiment 3, 100 unique prompts were sent to the models repeatedly until the entire dataset was exhausted in order to assess how the models performed when given large sets of unique prompts. Table 4 presents the results of the three experiments. In Exp 1, ChatGPT 3.5 has a slightly better performance with the highest F1-score (30.99%); F1-score is crucial as it balances recall and precision, providing a comprehensive measure of the model’s performance. Although better accuracy and recall are achieved by ChatGPT 3.5 and Gemini (30%), Perplexity is better at minimizing false positives with its highest precision (38.90%). In Exp 2, Perplexity performs better, with the highest F1-score (26.29%), accuracy (28%), and recall (28%). Because it provides a comprehensive measure of the model’s performance by balancing recall and precision, the F1-score is especially significant. ChatGPT 3.5 is better at reducing false positives with the highest precision, while Gemini has the lowest performance. In Exp 3, ChatGPT 3.5 has better accuracy, precision, and recall, followed by Gemini and Perplexity. Although the ChatGPT model may have trouble striking a balance between minimizing false positives and identifying true positives, the model’s relatively low F1-score suggests that there may be an imbalance between precision and recall.

Although LLMs have a broad range of knowledge, they may not be specialized in diagnosing complicated medical conditions like ML models that have been specifically trained in this area. The low F1-score in Table 4 may be related to LLMs’ limitations in handling medical diagnosis tasks, particularly diseases with similar or overlapping symptoms. The three experiments were carried out to test how the LLMs perform in different scenarios. Exp 1 tests the consistency and reliability of the LLMs in diagnosing diseases when a single prompt is used at a time. Exp 2 tests the LLMs’ capacity to manage more inputs concurrently because healthcare systems frequently handle several cases at the same time. Exp 3 tests the LLMs’ capacity to identify illnesses across a larger dataset through repeated exposure to various inputs. ChatGPT is an innovative technological tool for comprehending and processing natural language, making it suitable for interpreting and summarizing complex up-to-date information. Gemini is an adaptable tool that can handle various data types such as images and text, making it suitable for diagnostic purposes. Perplexity is specialized in comprehending and generating complex queries as well as maintaining context that can be vital for the retrieval and analysis of medical research. These LLMs lack specialized knowledge and are also capable of producing inaccurate answers, which can be critical in a medical context. They require high computational power to generate and process responses, which could limit real-time systems. Data security and patient privacy are concerns when handling sensitive medical data and they require proper validation and regulatory approval before they can be trusted and adopted for clinical use. To facilitate healthcare professionals’ comprehension of the reasoning behind a diagnostic output, LLMs integrate and analyze large amounts of medical data and produce human-readable explanations for their decisions.

The overall ML models’ performance in the study was moderate, suggesting the need for a sufficient dataset to enhance the diagnostic models. While the traditional SMOTE aided in balancing the dataset, employing an advanced oversampling method may help in improving the model performance. Even with GridSearchCV, the hyperparameters might still be improved, particularly for SVM. Better configurations could result from investigating alternative parameter tuning techniques like RandomizedSearchCV or Bayesian Optimization. To improve the results of the LLMs, the LLMs will be fine-tuned with a larger dataset, and an ensemble method will be employed to combine the strengths of different LLMs.

To integrate ML, XAI, and LLM techniques into an app, we propose two methods.

Method 1: Separate Training and Validation for ML and LLM
1. Train, test, and validate an ML model to diagnose malaria and typhoid based on the patient dataset
2. Apply LIME to explain the ML models’ diagnoses and how each symptom contributed to the diagnoses
3. Train, test, and validate an LLM model independently for generating explanations based on the patient dataset
4. Integrate the outputs from ML, LIME, and LLM to provide a comprehensive and interpretable diagnosis.

The advantage of method 1 is that it might lead to higher diagnostic performance considering the specific training of the two models (ML and LLM) for this task. The disadvantage is that the training and validation process of two independent models would increase the computational complexity of the diagnostic system, especially in combining the results to ensure consistency and coherence.

Method 2: Integrated ML, LIME, and LLM Process
1. Train, test, and validate an ML model to diagnose malaria and typhoid based on the patient dataset
2. Apply LIME to explain the ML models’ diagnoses and how each symptom contributed to the diagnoses
3. Use LLM for further explainability by passing the patient symptoms and ML results (with LIME explanations) through the model to generate diagnostic explanations in natural language.

The advantage of method 2 is its simplicity because an integrated pipeline reduces complexity, making the system easier to develop, test, and maintain, which we have implemented. Performance will be increased and computational overhead could be decreased by streamlining the procedure into a single pipeline. The explanations produced by LIME are directly considered by the LLM, which results in more logical and contextually appropriate explanations. The disadvantage is that the quality of the initial ML and LIME outputs determines the quality of the explanations provided by the LLM.

**Figure 14:**
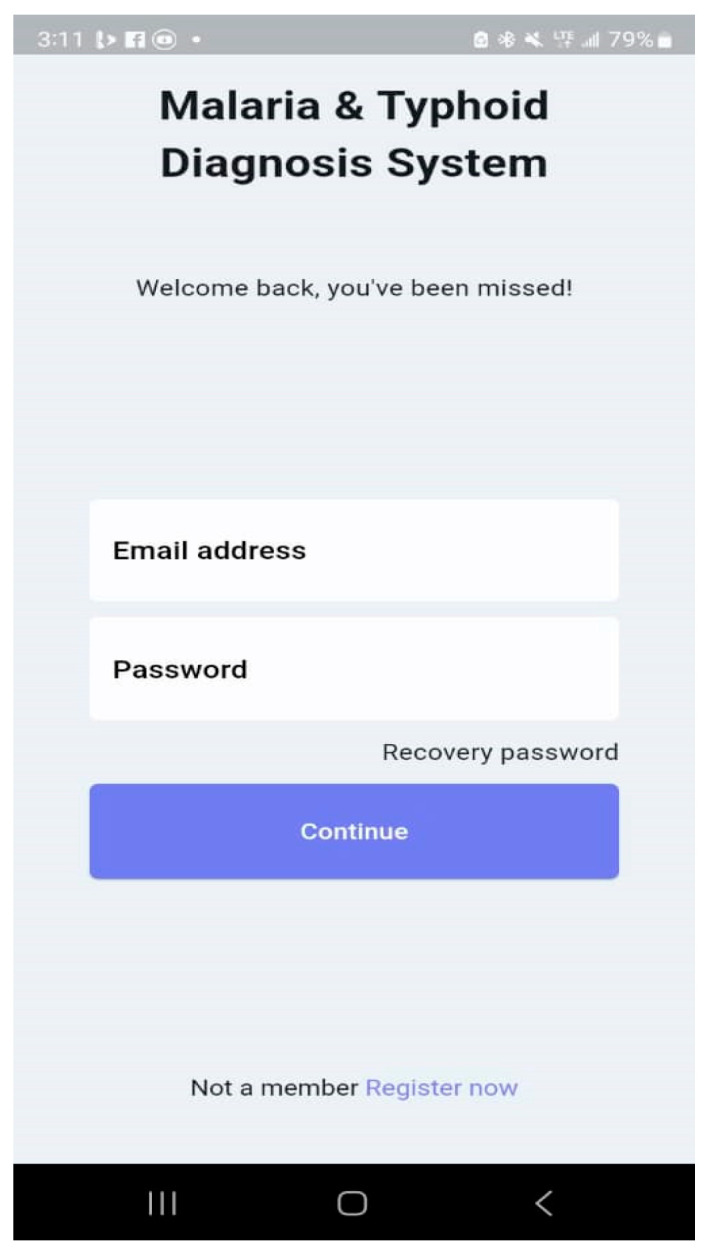
User Login.

**Figure 15:**
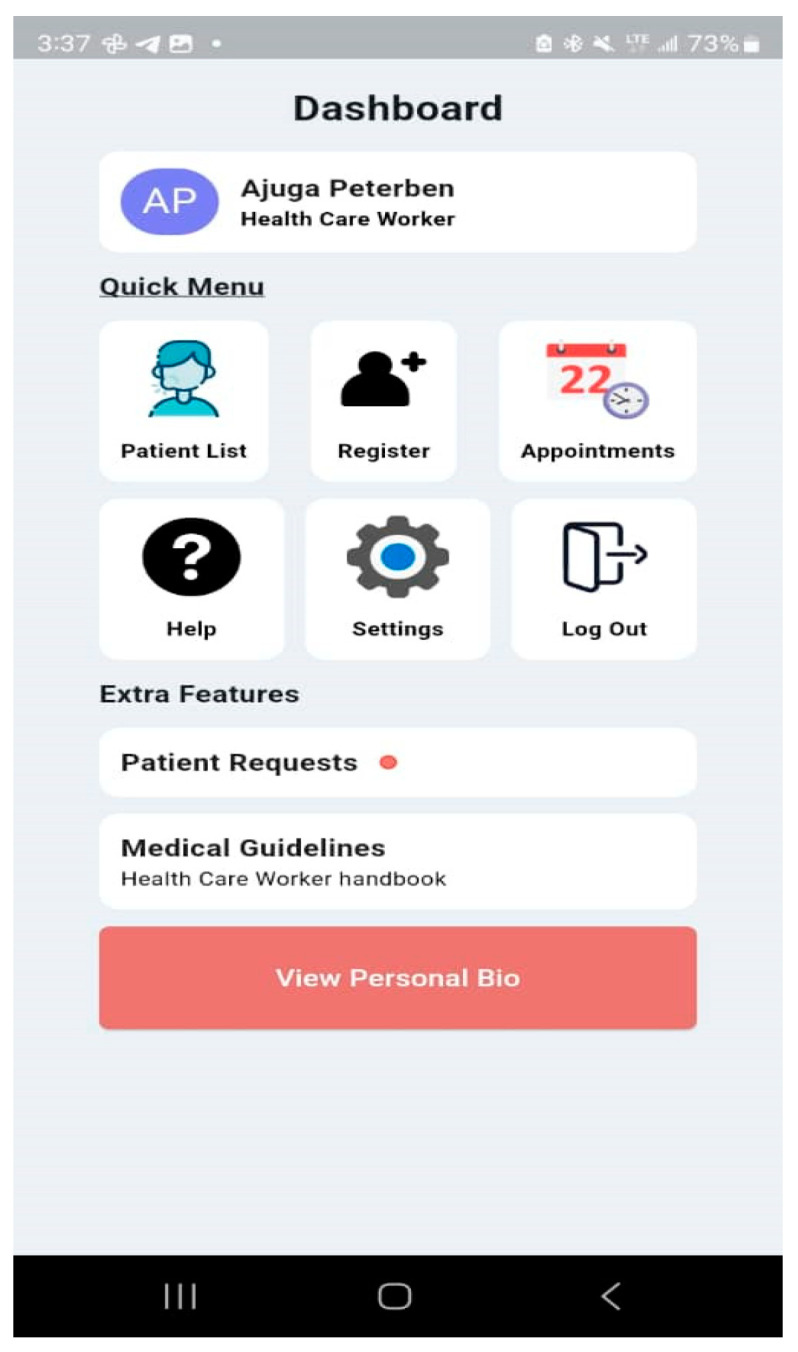
User Main Dashboard.

**Figure 16:**
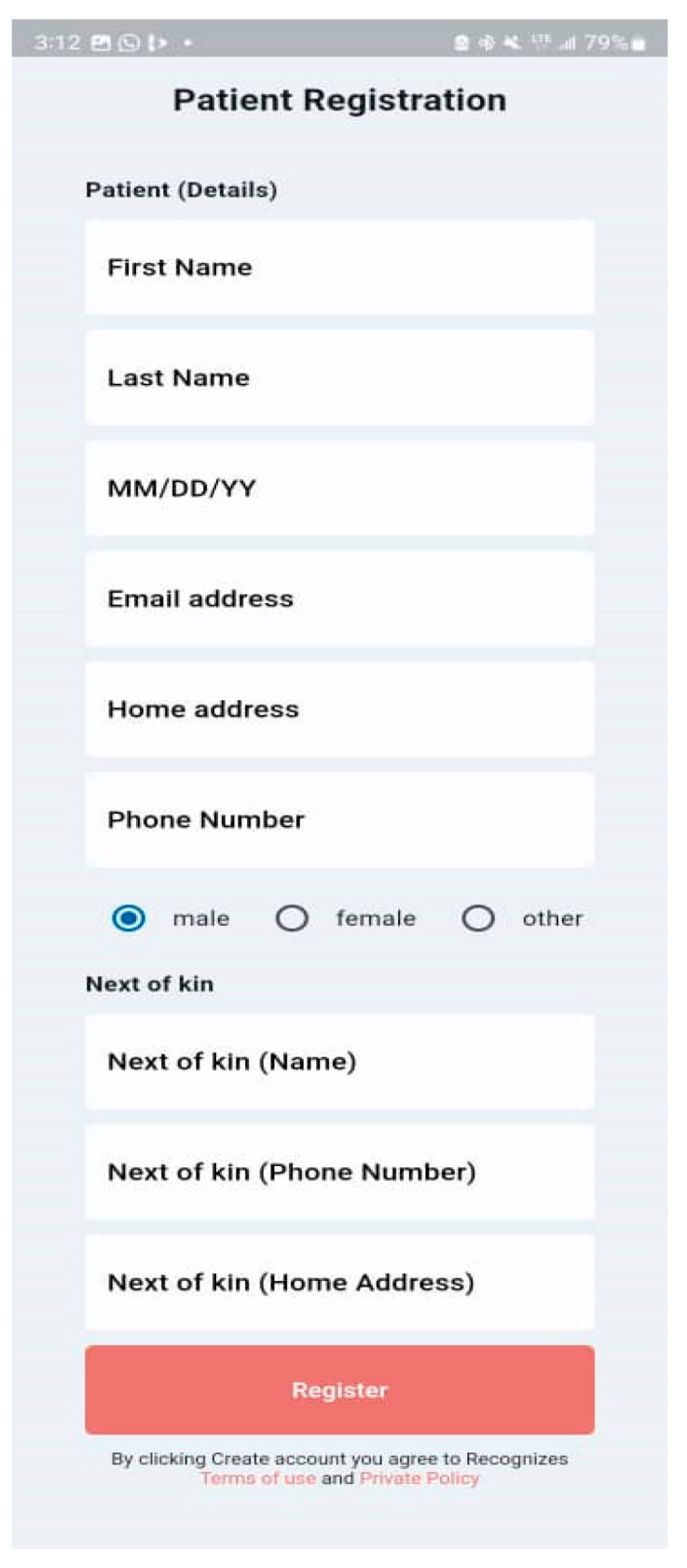
Patient Registration.

**Figure 17:**
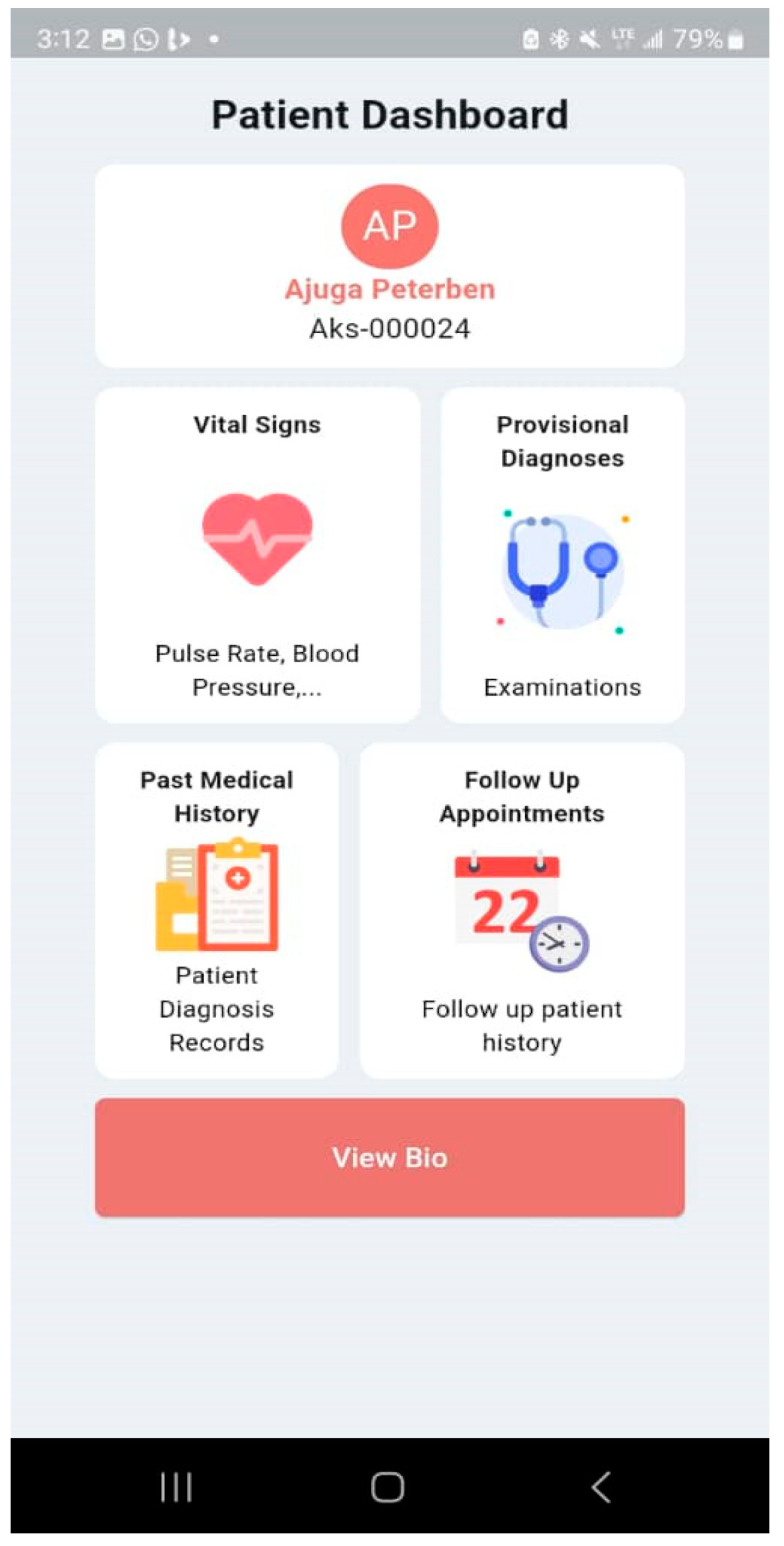
Patient Account Dashboard.

**Figure 18:**
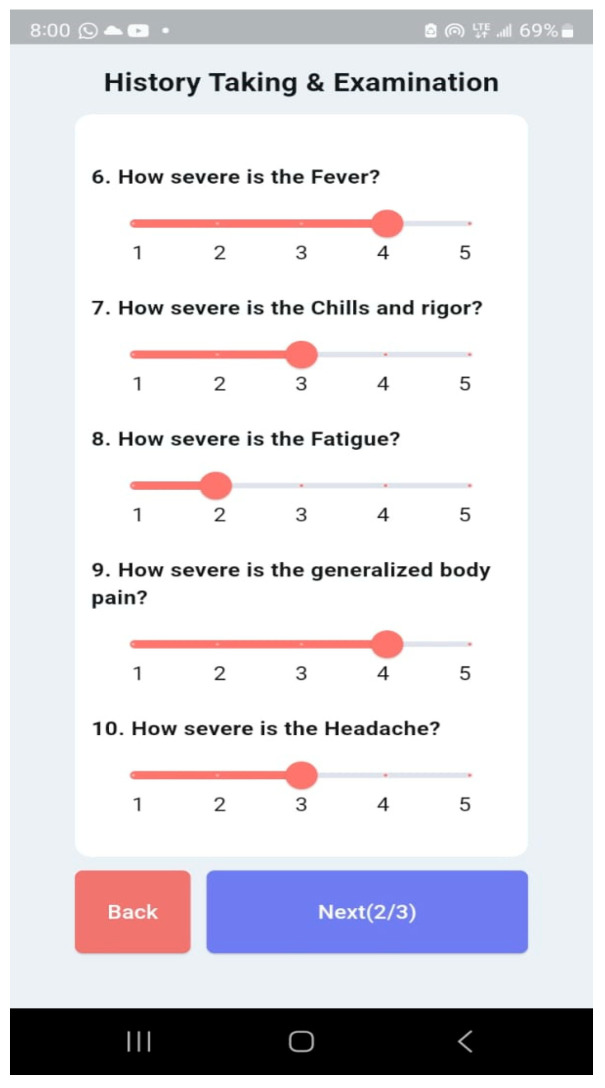
History Taking and Examination.

**Figure 19:**
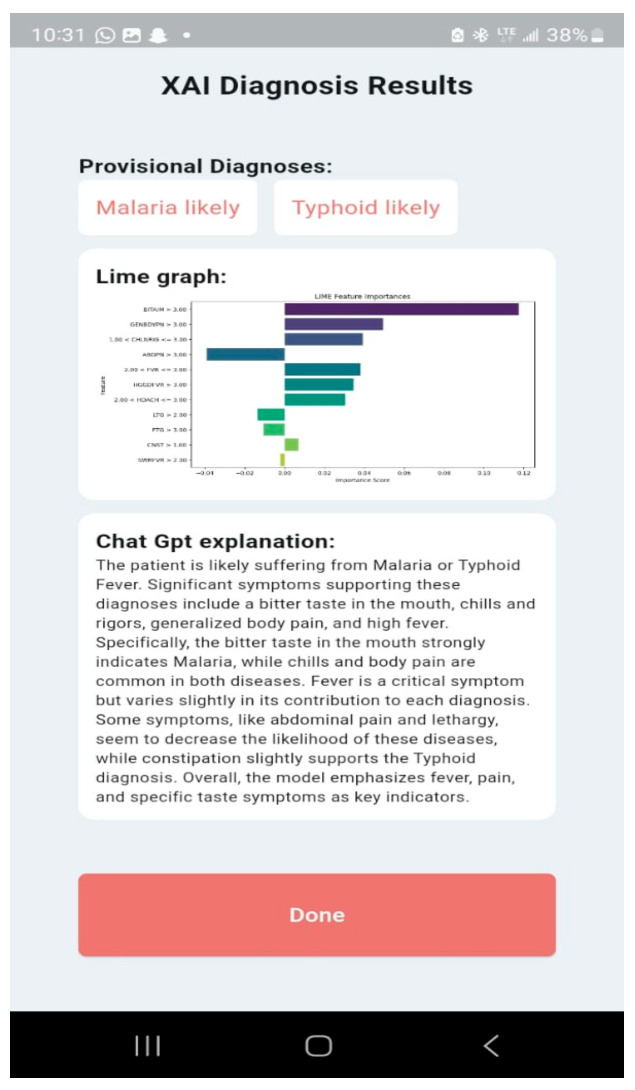
XAI Diagnosis Results.

The Malaria and Typhoid Fever Diagnosis System is a mobile app that healthcare workers can use to diagnose typhoid fever and malaria with an easy-to-use interface. The system comprises user authentication, a User main dashboard, and a Patient dashboard. The basic app requirement is an Android OS version 4.0 and above, 4 GB RAM: 2 GB minimum, ROM: 8 GB minimum, Display Layout: Portrait, and Internet connection. The user login is shown in Figure 14 and the User main dashboard is shown in Figure 15. The healthcare worker can register a patient, view a list of patients, and set up appointments for patients. Figure 16 is the patient registration form while Figure 17 is the patient dashboard where the patient vitals can be entered as well as the history taking and examination in Figure 18. The patient’s provisional diagnosis with XAI results is shown in Figure 19 with the LIME plot displaying the symptoms and how they influenced the model’s decision and the explanation by the ChatGPT LLM.

Previous studies [20,55,56] applied ML models for diagnosing malaria and typhoid fever, though these studies lacked appropriate interpretability in the decision-making process, which often results in medical experts having difficulties in comprehending the reasoning behind diagnostic results. This study integrated ML, XAI, and LLM to enhance transparency and interpretability in the diagnostic processes that align with global healthcare goals. The use of LIME for feature importance analyses and ChatGPT for generating context-aware explanations have distinguished the present study. Several factors can contribute to the low performance scores in Table 4. These include: (1) the dataset used during the training is limited in size and diversity, affecting the models’ ability to generalize to unseen cases; (2) LLMs may require further fine-tuning and optimization, as the complexity of the diseases being diagnosed may overlap with other illnesses, thereby challenging the models to accurately differentiate between them. Furthermore, LLMs did not show high domain tolerance to the investigated illnesses, hence fine-tuning them on domain-specific data can significantly improve their performance.

## 4. Conclusions

This study creates a medical diagnostic framework for Malaria and Typhoid fever by integrating XAI, LLMs, and ML models. This approach aims to demystify the black-box nature of ML models, offering transparent insights into how each feature or symptom affects the diagnosis. The RF model showed superior prediction performance in terms of accuracy, recall, precision, and F1-score compared to XGBoost and SVM. The high recall and precision values in RF are crucial for accurately diagnosing these diseases and for making appropriate treatment decisions. However, XGBoost exhibited the lowest log loss and fastest computation time. Further analysis indicates that SVM performs worse than the other two models, making it less suitable for this dataset. The study suggests that ensemble techniques like RF and XGBoost better capture the complex relationships between symptoms and diseases. The XAI analysis identified BITAIM, CHNLNRIG, LTG, ABDPN, MSCBDYPN, FVR, GENBDYPN, FTG, and HGGDFVR as key features for predicting Malaria and Typhoid. Among LLMs, ChatGPT 3.5 performed slightly better than Gemini and Perplexity. This study has shown how RF, LIME, and GPT can be used effectively to diagnose typhoid fever and malaria using a mobile-based system that meets the crucial requirements of interpretability and transparency, improving the diagnostic process’s acceptability and understanding among medical professionals. Future research should examine the application of various machine learning models, XAI techniques, and LLMs on a variety of datasets and across other medical conditions, such as in the diagnosis of diabetes, cardiovascular diseases, and cancer detection, to further validate and generalize the findings of this study. The validity of AI-driven diagnostics can be strengthened by extending its application to additional medical conditions. This will ultimately improve patient outcomes in a range of healthcare domains.

## Figures and Tables

**Table 1:** Statistics of male and female patients in the dataset.

Patient Age (Years)	<5	5–12	13–19	20–64	≥65	Total
Male	534	346	150	1012	133	2175
Female	419	323	213	1605	135	2695
Total	953	669	363	2617	268	4870

**Table 2:** Symptoms and diseases with abbreviations.

Symptom/Disease	Abbreviation
Abdominal pains	ABDPN
Bitter taste in mouth	BITAIM
Chills and rigors	CHLNRIG
Constipation	CNST
Fatigue	FTG
Fever	FVR
Generalized body pain	GENBDYPN
Headaches	HDACH
High-grade fever	HGGDFVR
Lethargy	LTG
Muscle and body pain	MSCBDYPN
Stepwise rise fever	SWRFVR
Malaria	MAL
Typhoid fever/Enteric fever	ENFVR

**Table 3:** Diagnostics model performance.

Algorithm	Accuracy	Precision	Recall	F1-Score	Log Loss	Computation Time
XGBoost	0.7129	0.7056	0.7129	0.7066	0.7808	2 min, 32 s
RF	0.7199	0.7129	0.7199	0.7145	1.0548	14 min, 9 s
SVM	0.6860	0.6865	0.6860	0.6821	0.8016	1 h, 22 min, 7 s

**Table 4:** Large language models’ performance.

Experiment	Algorithm	Accuracy	Precision	Recall	F1-Score
Exp 1	Chat GPT 3.5	0.3000	0.35562	0.3000	0.30999
	Gemini	0.3000	0.3449	0.3000	0.2908
	Perplexity	0.2600	0.3890	0.2600	0.28736
Exp 2	Chat GPT 3.5	0.2600	0.2909	0.2600	0.2615
	Gemini	0.2700	0.2607	0.2700	0.2296
	Perplexity	0.2800	0.2524	0.2800	0.2629
Exp 3	Chat GPT 3.5	0.3297	0.3324	0.3297	0.2926
	Gemini	0.2895	0.2709	0.2895	0.2728
	Perplexity	0.2632	0.1957	0.2632	0.2171

## Data Availability

The raw data supporting the conclusions of this article will be made available by the authors on request.
